# The Influence Mechanism of Knowledge-Based Professionals’ Core Value Identity on Creativity From the Perspective of the Knowledge Economy

**DOI:** 10.3389/fpsyg.2021.724463

**Published:** 2021-10-22

**Authors:** Yilin Zhou, Chunyan Wang, Fakhar Shahzad, Majid Murad

**Affiliations:** ^1^School of Management, Jiangsu University, Zhenjiang, China; ^2^School of Finance and Economics, Jiangsu University, Zhenjiang, China; ^3^Department of Business Administration, ILMA University, Karachi, Pakistan

**Keywords:** core values identity, tacit knowledge transfer, individual creativity, knowledge workers, knowledge economy

## Abstract

In the era of the knowledge economy, tacit knowledge transfer is a key strategy for developing personal creativity, but it also is affected by core value agreement. Knowledge-based professionals have innate sensitivity with core value agreement. In the knowledge capital development system, tacit knowledge transfer is a key strategy to be discovered. This research investigates the core value identity mechanism of knowledge professionals from the perspective of the knowledge economy. The results revealed the intrinsic relationship between core values identity, tacit knowledge transferring, and personal creativity based on knowledge-based professionals’ data and employing core values identity, tacit knowledge transferring, and personal creativity based on knowledge-based professionals’ data and structural equation modeling. The results also represent the incentive path of tacit knowledge transferring in personal creativity under the core value agreement condition, which may provide a theoretical inspiration to foster knowledge-based talent creativity.

## Introduction

The creativity of knowledge-based workers or professionals originates from national innovation capability and is the core element of national competitiveness under the knowledge economy. In today’s world, every country pays great attention to cultivating knowledge-based professionals’ creativity and strives to take the initiative or maintain a leading position in international competition (Sobocka-Szczapa, 2020). Knowledge-based professionals are a group with a far-reaching influence on society’s development and have long attracted the state, the government, and various organizations. In the knowledge economy era, knowledge-based professionals contain a considerable amount of knowledge, which is the carrier of intellectual capital and leads the development direction of the times (Chen S.Y., 2012; Gaviria-Marin et al., 2018). Meanwhile, knowledge transfer among knowledge-based professionals serves as a foundation for economic and political deductive models such as ideological communication, technological transfer, and product innovation. At the same time, there is a significant difference between the intellectual talents in modern society and those in the traditional mode, and they are concerned with their interests and the social realization of their lifetime value (Gao et al., 2008). They also care about individuals, teams, and organizations, society’s progress, core values and are profoundly interwoven into their own ideas and activities.

The identification of core values is a significant issue in the spiritual world for knowledge-based professionals. It is no less than the influence of material interests, and this is one of the personality traits (Chen and Edgington, 2012; Li et al., 2020) of knowledge-based professionals. The core values reflect the path of socialism, represent the direction of society, bear the people’s destiny, and are rich in content. The study of Fei (2011) demonstrates that there are objectively intertwined situations in which all kinds of thoughts and values are intertwined in our society. Mucai (2011) believes that “loyalty, filial piety, love, faith, and peace” are the core values advocated during the Republic of China’s modern period. It inherited an excellent moral spirit, but it failed to integrate it with the socialist age trend. Jiyan (2012) believes that socialism’s core values are a theoretical system with rich connotations, but the vitality lies in the masses and needs to be transformed into people’s conscious pursuit. Meanwhile, Chen B. (2012) argued that socialism’s core values should include humanism, benevolence, democracy, justice, and harmony. In addition, the socialist core values derived from the socialist core value system are stated in particular as co-constructing, sharing, equal rights, emancipation, friendship, harmony, progress, and prosperity (Du, 2012).

In the era of a knowledge economy, tacit knowledge transfer is the key to promoting knowledge-based professionals’ intellectual development, but the identification of core values restricts it (Bjorvatn and Wald, 2020). Tactic knowledge transfer research is garnering considerable attention within the knowledge capital development system of knowledge-based professionals. The study of Cabrera and Cabrera (2005) explored the knowledge transfer mechanism of knowledge workers and emphasized the role and function of tacit knowledge transfer in the process of employee creativity. The results from the study of Bock et al. (2005) described the mechanism of knowledge transfer and knowledge sharing in knowledge-based teams, emphasizing that tacit knowledge transfer is a key strategy for the cultivation of knowledge workers’ creativity. Lei et al. (2006) analyzed the connotation of knowledge workers from the perspective of tacit knowledge transfer and believe that knowledge workers are engaged in the creation, exchange, sharing, and development of various types of knowledge in various types of enterprises and can create greater value for their organizations or enterprises. Prior study suggests a positive relationship between knowledge sharing and personal creativity, comprehensive research on the subject is lacking. Meanwhile, knowledge employees represent the enterprise’s most scarce and valuable capital; nonetheless, the hidden capital and motivation of knowledge workers are difficult to standardize across individuals (Yanhua and Shi, 2007; Zhang and Gu, 2016).

In the existing research framework of intellectual talent creativity, intellectual talents’ thoughts and behaviors are separated (Bujor and Avsilcai, 2016; De Silva et al., 2018). Political science and sociology focus on the ideology of intellectual talent, ignoring personal creativity, while economics and management focus on profit-driven creative growth paths while ignoring the core value identity of the origin of creative thinking. Intellectual talents are the unity of “meaning” and “doing.” They have a high degree of consistency in thought and behavior. One-sided research can only aggravate the ambiguity of knowledge talents. Therefore, it is a practical method and approach to exploring intellectual talents’ creativity from the origin of core value identity. This study attempts to investigate the impact of the promotion mechanism of knowledge-based professionals’ core value identity on creativity, which has not been discussed previously.

## Materials and Methods

### Basic Theoretical Analysis and Underpinning

#### Core Value Identification

Marxism believes that the essence of value is a relationship between the subject and the object that satisfies the subject’s needs, reflecting the object’s influence on the subject. The left and right directions are from the object to the subject, and the values are the basic views of the subject and the object. They are the essential viewpoints of the value target, status, and realization. They are the relatively stable positions held by people when dealing with various value issues. The left and right directions are from the subject to the object (Hongwen, 2016). Whether people can realize or feel, values are objective, and values exist in the human mind (Steyn and Du Toit, 2009). Therefore, values are the basis for the generation of values, not the product of values.

The core values are the core ideas, spirits, and goals of the ruling party and reflect the ruling party’s essential motives. In addition to traditional Marxist principles, the socialist core values include concrete socialist goals with contemporary Chinese features, the spirit of the times, and socialist reform and creativity. The four aspects of the concept of honor and disgrace are the “people-oriented” values, which embody serving the people wholeheartedly. The recognition of core values by knowledge-based professionals includes two fundamental elements: emotional identity and behavioral identity. Here, emotional identity refers to emotionally cherishing core values, and behavioral identity refers to supporting core values in action.

#### Tacit Knowledge Transfer

Knowledge capital consists of tacit knowledge and explicit knowledge as defined by Nonaka (1994). Explicit knowledge is the knowledge that can be clearly expressed, communicated, and transmitted. Tacit knowledge is implicit in thinking and cannot be described by words, numbers, or symbols. Explicit knowledge is well-documented and widely recognized, whereas tacit knowledge is gained via hands-on experience and is only partially recorded (Nonaka, 1994). In knowledge capital, tacit-knowledge is the main body, occupying about 80% of the total knowledge capital, and many valuable human spiritual treasures also exist in the form of tacit knowledge (Polanyi and Sen, 2009). Resources-Advantage (R-A) theory supports the premise that tacit knowledge transfer is a vital mechanism for developing a sustainable competitive advantage over the long term (Hunt and Morgan, 1995). It describes the mechanisms by which some firms can outperform their competitors consistently. Tacit knowledge is a precious resource since it differs from explicitly acquired knowledge. Moreover, the proportion of explicit knowledge in intellectual capital is low, and it is the “corner of the iceberg” of intellectual capital. The knowledge that has a substantial promotion function for social development is tacit (Rosellini and Hawamdeh, 2020).

Modern network organizations that wish to be innovative and gain a competitive edge must discover a means to capture this resource, which they do not own, and change it from tacit to explicit. Because explicit knowledge can be codified, it is easily transferable. Tacit knowledge is difficult to codify or document therefore it is learned through meaningful practical experience (Kucharska and Rafał, 2016). The process of knowledge creation begins with the generation and dissemination of tacit knowledge, which is generated from socialization, experience facilitation, and individuals’ ability to engage with their coworkers (Astorga-Vargas et al., 2017). Additionally, knowledge sharing has been ingrained in firms’ business strategy, assisting them in growing and innovating in the market and gaining a competitive edge (Ganguly et al., 2019). Recessive knowledge transfer refers to the transfer of existing tacit knowledge capital from one knowledge subject to another, significantly different from explicit knowledge transfer (Nonaka, 2009). Among the various strategies for intellectual capital development, knowledge transfer has received the most attention. Nowadays, the way tacit knowledge is considered places a premium on its organizational importance.

In comparison to explicit information, tacit knowledge is unique, and as a result, helpful to businesses (Kucharska and Rafał, 2016). Numerous studies have been conducted on a variety of critical business themes, including tacit knowledge transfer inside multinational organizations (Guo et al., 2020); joint ventures (Park et al., 2012); sales and marketing (Arnett et al., 2021); and strategic alliances (Qiu and Haugland, 2019). For knowledge-based professionals, tacit knowledge transfer can be divided into three primary forms: tacit knowledge transfer with colleagues, tacit knowledge transfer between teams, and tacit knowledge transfer with society. In this study, they are called “X-direction knowledge transfer,” “Y-direction knowledge transfer,” and “Z-direction knowledge transfer.” The author quantified the relationship between tacit knowledge transmission and personal creativity to accomplish the current study objective.

#### Personal Creativity

Personal creativity is the soul of knowledge-based professionals. The theory of personal creativity focuses on the individual (Runco, 2020). The study of personal creativity should be carried out from psychological intelligence to penetrate the essence of personal creativity. In this perspective, social recognition and attributions by some audiences are intrinsically tied to creativity (Runco and Beghetto, 2019). In 1996, based on “three-dimensional intelligence theory,” Sternberg put forward the famous “successful intelligence theory” through long-term investigation and analysis and believed that human intelligence includes analytical intelligence, creative intelligence, and practical intelligence, while creative intelligence is the key element (Zhang and Sternberg, 2002). Analytical intelligence is the capacity to analyze, comprehend, and evaluate unfamiliar areas to find numerous answers to issues; creative intelligence is the capacity to identify shortcomings, transcend the present, overcome constraints, and produce new thinking activities (Silva, 2018).

The ability to use existing tools, methods, hobbies, and interests to create better ideas; practical intelligence refers to the ability to flexibly apply existing ideas, methods, and skills to solve real-world problems (Herrmann and Felfe, 2014). They were transforming into practical activities, translating ways of thinking into real products, and turning experience into innovation. Personal creativity theory asserts that individuals generate unique and successful interpretations while acknowledging goals and discretion (Runco, 2020). The owner’s abilities to create, store, and articulate tacit information are also contingent on his or her personality, dexterity, explicit knowledge, and access to external sources of formal knowledge. Personal creativity is inextricably linked to one’s cognitive preferences (Kim et al., 2011). Additionally, they rely on the capacity to build on one’s own and other people’s experiences.

Using a sender-recipient model and arguments from learning theory and transaction costs economics, Steinberg et al. (2021) argued that emerging market multinational enterprises benefit more from and are thus more likely to engage in mechanisms that increase individual capability and motivation. It described that the stronger one’s aptitude to impart, absorb, and transfer knowledge, the more likely to a tacit knowledge transfer. Aside from restructuring and effective transfer of knowledge, skills, and information, knowledge sharing also includes the development of creative knowledge and innovative ideas (Lee, 2018). In terms of effectiveness, the greater an individual’s creativity, the more effective his or her own creative work (Stoykov, 2021). Meanwhile, a high degree of tacit knowledge transfer fosters individual learning processes, and as a result, enhances an individual’s creative skills, which is another component of personal creativity.

### Proposal of Research Hypothesis

#### Analysis of the Promotion Effect of Knowledge-Based Professionals’ Core Values Identity on Tacit Knowledge Transfer

##### Analysis of the promotion effect of emotional identity on tacit knowledge transfer

Emotional identity means that knowledge-based professionals have a high degree of recognition for core values’ ideas, viewpoints, and implementation methods (Currie et al., 2015). In the knowledge economy era, knowledge-based professionals are continuously implementing knowledge transfer, especially tacit knowledge transfer. However, knowledge-based talents are emotional economic individuals who focus on their interests and who focus on their interests and have a high degree of sensitivity to core values and life (Burnette, 2017). If they share the basic principles, they will invariably experience spiritual fulfillment, future trust, a willingness to connect directly with colleagues, get along well with colleagues, care about society, care about the team’s growth, and share their knowledge with others (Qiu and Haugland, 2019). A positive mental state is very suitable for the transfer of tacit knowledge, and it is not easy to make a substantial contribution to the transfer of tacit knowledge.

On the contrary, if knowledge-based talents resist core values and believe that the future is bleak, they must violate their moral norms to achieve their goals, lack passion for life, and only struggle to cope with direct knowledge transfer.

Based on the above analysis, the following research hypotheses can be proposed:

**H1a:** The emotional identity of knowledge-based professionals in China is positively correlated with X-knowledge transfer.
**H1b:** The emotional identity of knowledge-based professionals in China is positively related to Y-knowledge transfer.
**H1c:** The emotional identity of knowledge-based professionals in China is positively related to Z-knowledge transfer.

##### Analysis of the promotion effect of behavioral identity on tacit knowledge transfer

Behavioral identity is the support and obedience of knowledge-based professionals to the core values in concrete actions. Behavioral identity results from further deepening of emotional identity, but they both impact the work behaviors. Without a professional identity, access to tacit knowledge and membership in a professional network is hampered. As a result, a risky position is devoid of the tacit and particular knowledge required for success (Posukhova and Maskaev, 2016). Knowledge-based professionals not only emotionally focus on the core values but also actively participate in core values activities. Knowledge-based professionals’ spiritual world still transmits the promotion of tacit knowledge transfer by behavioral identity (Nonaka et al., 2000). Suppose the intellectual talents have a behavioral identity with the core values. They will then develop a dynamic of physical and mental contact in promoting, studying, and distributing essential principles, resulting in a lively spiritual environment free of ideological disorders. Be able to concentrate on work and career. Such a state of mind must be conducive to in-depth communication with colleagues, superiors, and subordinates (Burnette, 2017). On the other hand, if people act in ways that are antithetical to basic principles, it costs personal time and energy, impedes the exchange and transmission of tacit information, and even results in severe occurrences that contribute to personal spirit decay (He et al., 2013; Abdelwhab Ali et al., 2019).

Based on the above analysis, the following research hypotheses can be proposed:

**H2a:** The behavioral identity of knowledge-based professionals in China is positively related to X-knowledge transfer.
**H2b:** The behavioral identity of knowledge-based professionals in China is positively related to Y-knowledge transfer.
**H2c:** The behavioral identity of knowledge-based professionals in China is positively related to Z-knowledge transfer.

#### Analysis of the Promotion Effect of Tacit Knowledge Transfer of Knowledge Talents on Personal Creativity

In the era of the knowledge economy, personal creativity is essentially a kind of intellectual ability. Creativity requires numerous resources, such as time, materials, teamwork, much hard work, information resources, and hard mental energy (Shalley et al., 2004). Knowledge is a key resource for personal creativity and sharing knowledge and information among employees is a vital source of personal creativity. The psychological intelligence of knowledge-based professionals is also the embodiment of knowledgeability, especially in controlling tacit knowledge (Lee, 2018). Knowledge-based professionals’ tacit knowledge transfer is essentially technical exchange, experience exchange, and exchange of ideas. It can trigger personal inspiration, expand personal knowledge, and deepen individual thinking. The more efficient tacit knowledge transfer is, the more an individual’s knowledge capital is, the stronger the ability is, and the more conducive to personal creativity development (Men et al., 2019).

Most importantly, knowledge sharing leads to organizational innovation and creativity, as it involves reorganizing and successfully transmitting information and generating new knowledge and imaginative ideas (Cabrera and Cabrera, 2005). In a real society, tacit knowledge transmission must occur in various forms, including enhancing collaboration among colleagues, strengthening teamwork, and learning from society by absorbing the essence of social growth (Burnette, 2017; Guo et al., 2020). It can be observed that tacit knowledge transfer and individual innovation are intrinsically linked. Any form of tacit knowledge transfer is indispensable in cultivating personal creativity, and it is also irreplaceable for explicit knowledge transfer.

Based on the above analysis, the following research hypotheses can be proposed:

**H3a:** X is positively related to knowledge transfer and personal creativity.
**H3b:** Y is positively related to knowledge transfer and personal creativity.
**H3c:** Z is positively related to knowledge transfer and personal creativity.

### Factor Decomposition

#### Factor Decomposition of Core Values

According to the connotation of core values and the investigation of knowledge-based professionals’ psychology and behavior, knowledge-based professionals’ core values can be divided into two elements: emotional identity and behavioral identity.

The elements of emotional identity are decomposed into four indicators: ① Satisfied with the ideas and ideas advocated by mainstream values; ② That mainstream values reflect the spirit of freedom, equality, and justice; ③ That the ruling party is saying words and deeds in the promotion of mainstream values consistent; ④ Believe that mainstream values are in line with China’s development path.

The behavioral identity factor is decomposed into four indicators: ① To resist social corruption; ② To actively learn the essence of mainstream values; ③ To actively promote mainstream values’ outline, content, and value; ④ To resent the Western “universal value” and opposing attitudes.

#### Factor Decomposition of Tacit Knowledge Transfer

Based on the tacit knowledge transfer theory and related research results, the factor decomposition of X-direction knowledge transfer, Y-direction knowledge transfer, and Z-direction knowledge transfer can be carried out. X decomposes the knowledge transfer elements into four indicators: ① The desire to cooperate with colleagues and mutual help; ② Good at finding the right time and deep communication with colleagues; ③ Good at grasping the way of communication with colleagues; ④ Good at discovering the most the content of exchange of values.

Y decomposes knowledge transfer into four indicators: ① Actively participates in various group academic activities; ② Often organizes or assists various academic seminars; ③ Is good at absorbing the industry’s mature research results; ④ Is good at public expressing their ideas.

Z to knowledge transfer is decomposed into four indicators: ① Actively promote their technology or ideas to the society; ② Actively participate in various social activities; ③ Profoundly think about social events related to their profession; ④ Actively conduct social surveys explore the needs and direction of society.

#### Factor Decomposition of Personal Creativity

According to the successful intelligence theory and related research results, the factor decomposition of personal creativity can be carried out, including the following four indicators: ① Has the ability to analyze problems; ② Has particular exploration, trial and error, and adventurous spirit; ③ Is good at transforming technology for the product, or to transform the mind into a spirit; ④ Good at flexible application of the knowledge, skills, and experience in daily work (Mei et al., 2015).

### Establishment of Research Model

**FIGURE 1:**
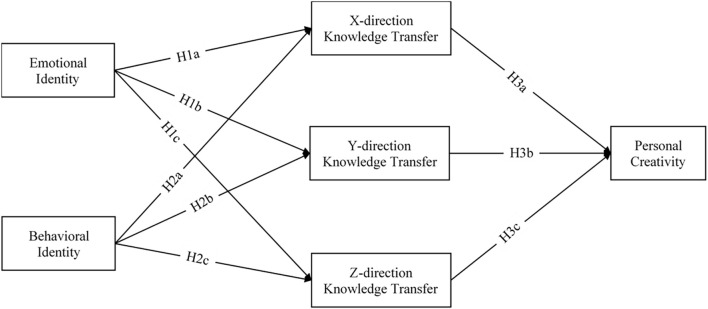
Proposed research model.

Based on the research hypothesis’s characteristics, a structural equation model (SEM) test is proposed in this study. The structural equation model is divided into three categories: one is pure verification, that is, the authenticity of the test model; the second is the selection model, that is, selecting one optimal model from multiple proposed models; the three types are generating models, that is, construct an effective model based on existing conditions. This study’s application is purely verification; only a model derived from theory is used to fit a set of existing sample data. The purpose of the analysis is to verify whether the designed model fits into the existing sample data. So psychologically decide to accept or reject this model. According to the research hypothesis and factor decomposition content, the research model is shown in Figure 1.

## Results and Discussion

### Data Collection

This data survey collects data from knowledge-based professionals in various organizations in China. The data evaluation standard is the Likert 7-point scale. According to the content of factor decomposition, the design of the measurement items of core value identification, tacit knowledge transfer, and personal creativity was firstly designed, and 24 items were designed. This study’s data survey began on January 6 ended on February 16, 2021, for 41 days and obtained 200 valid samples of knowledge talents. The sample characteristics are shown in Table 1.

**TABLE 1:** Sample characteristics.

Feature	Category	Number of samples	Proportion	Feature	Category	Number of samples	Proportion
Gender	Male	120	60	Job title	Senior title	54	27
	Female	80	40		Intermediate title	78	39
Industry	Colleges and universities	70	35		Junior title	62	31
	Medical unit	64	32		No title	6	3
	Research institute	32	16	Education	Doctor	70	35
	State-owned enterprise	24	12		Master’s degree	70	35
	Private enterprise	10	5		Bachelor	38	19
Distribution area	North China	16	8		Specialist and below	22	11
	South China	24	12	Length of service	<5 years	16	8
	Southeast region	34	17		5–10 years	28	14
	Central Plains	36	18		11–15 years	44	22
	North-east area	16	8		16–20 years	35	18
	North-west region	18	9		21–25 years	33	16
	Southwest region	24	12		26–30 years	24	12
	Huadong region	32	16		>30 years	20	10
Age	<30 years old	24	12	Position	Division level and above	24	12
	31–35 years old	36	18		Section level	34	17
	36–40 years old	32	16		The masses	142	71
	41–45 years old	44	22	Profession	Science and engineering	60	30
	46–50 years old	30	15		Agriculture and forestry	18	9
	50–55 years old	20	10		Medicine	34	17
	>55 years old	14	7		Literary history philosophy	28	14
Political background	Communist party members	66	33		Law and politics	14	7
	Democratic party	40	20		Economy and management	32	16
	Non-partisan	94	47		Other	14	7

### Reliability Test

Reliability is the reliability of the measurement scale and is one of the scale’s quality measures. If various surveyors use the scale to measure the same type of measuring object at different times and locations and the findings are essentially the same, the scale is reliable. In conventional statistical analysis, the standard method of reliability testing is exploratory factor analysis. In this study, based on the sample of 200 knowledge-based professionals using SPSS version 26 software, the measured metrics’ reliability test results are shown in Table 2. The measurement scales designed by the Institute have good reliability.

**TABLE 2:** Reliability test results.

Element	Maximum load value	Minimum load value	Minimum CITC value	Cronbach α value	Cumulative interpretation
Emotional identity	0.781	0.456	0.57	0.8901	71%
Behavioral identity	0.782	0.400	0.51	0.7129	59%
X-direction knowledge transfer	0.759	0.444	0.51	0.7349	44%
Y-direction knowledge transfer	0.838	0.370	0.49	0.7254	56%
Z-direction knowledge transfer	0.824	0.314	0.43	0.8019	43%
Personal creativity	0.878	0.458	0.60	0.7610	52%

### Validity Test

Validity is the authenticity of the measurement scale and is one of the measures of scale management. In some cases, the scale designer cannot fully express his or her own measurement intention, causing the measured person to be ambiguous about the scale so that the scale’s validity is poor. A standard method of validity testing is confirmatory factor analysis (CFA). In this study, the validity test results of the measured metrics are shown in Table 3. The results show that the measurement scales designed in this study have good validity.

**TABLE 3:** Validity test results.

Scale name	χ^2^/*d.f.*	RESEA	Maximum load value	Minimum load value	Minimum *T*-value
Emotional identity	1.64	0.033	0.68	0.33	3.22
Behavioral identity	1.53	0.029	0.72	0.29	4.18
X-direction knowledge transfer	1.11	0.020	0.64	0.31	4.66
Y-direction knowledge transfer	1.21	0.013	0.60	0.22	5.09
Z-direction knowledge transfer	2.34	0.027	0.61	0.28	7.77
Personal creativity	2.65	0.014	0.55	0.27	5.10

Table 4 represents the values of items loadings, composite reliability (CR), and average variance extracted (AVE) to confirm the validity. The cutoff values for item loadings and CR have been proposed as above 0.7 (Fornell and Lacker, 1981; Henseler et al., 2014). Results from table shows that the item loadings are ranging from 0.717 to 0.889, and the values of CR are ranging from 0.827 to 0.923, higher than the recommended value. The AVE is the ratio of the variance of the indicator variables that can be explained by the potential variables and is an indicator of convergent validity, and the general criterion is that the AVE is greater than 0.5 (Fornell and Lacker, 1981; Henseler et al., 2014). From Table 4, it can be observed that the AVE values are greater than the recommended value, which indicates that the study variables can be distinguished from each other.

**TABLE 4:** Results of factor loadings, composite reliability (CR), and AVE.

Variable	Items	Loadings	CR	AVE
Emotional identity	EI1	0.810	0.898	0.688
	EI2	0.871		
	EI3	0.841		
	EI4	0.793		
Behavioral identity	BI1	0.889	0.921	0.745
	BI2	0.799		
	BI3	0.867		
	BI4	0.895		
X-direction knowledge transfer	X1	0.887	0.916	0.732
	X2	0.852		
	X3	0.866		
	X4	0.815		
Y-direction knowledge transfer	Y1	0.836	0.908	0.712
	Y2	0.809		
	Y3	0.875		
	Y4	0.853		
Z-direction knowledge transfer	Z1	0.736	0.827	0.545
	Z2	0.717		
	Z3	0.758		
	Z4	0.741		
Personal creativity	PC1	0.878	0.923	0.749
	PC2	0.849		
	PC3	0.857		
	PC4	0.877		

Furthermore, Table 5 represents the inter-construct correlation among the variables to confirm the validity. As suggested by Fornell and Lacker (1981), “the average variance shared between a construct and its measures should be greater than the variance shared by the construct and any other constructs in the mode.” Table 5 represents the square root of the AVE values greater than the correlation coefficient between the variables, indicating no discriminant validity issue.

**TABLE 5:** Factor correlation coefficient matrix.

Factors	Emotional identity	Behavioral identity	X-direction knowledge transfer	Y-direction knowledge transfer	Z-direction knowledge transfer	Personal creativity
Emotional identity	**0.829**					
Behavioral identity	0.656	**0.863**				
X-direction knowledge transfer	0.545	0.458	**0.856**			
Y-direction knowledge transfer	0.515	0.556	0.476	**0.844**		
Z-direction knowledge transfer	0.487	0.492	0.512	0.485	**0.738**	
Personal creativity	0.523	0.534	0.595	0.536	0.472	**0.865**

*Diagonal bold-face values show the square root of AVE.*

The CFA has been applied to assess the measurement model’s overall goodness of fit. The values of Chi square/degree of freedom (CMIN/DF) = 1.316, RMSEA = 0.026, CFI = 0.959, NFI = 0.942, IFI = 0.922, and GFI = 0.922 indicating the goodness of model fit.

### Model Verification

According to the reliability test and validity test results, the questionnaire has good reliability and validity, and the sample data has high reliability and validity, which can be included in the research model for statistical analysis. The reliability and validity tests of the measurement scale and the whole model were tested using SPSS 18.0 software and LISREL 8.7 software. The test results are shown in Table 6.

**TABLE 6:** Effect matrix.

External variable	Endogenous variable	Hypothesis	Coefficient load	Standard error	*T*-value
Emotional identity	X-direction knowledge transfer	H1a	0.46	0.11	4.11
Emotional identity	Y-direction knowledge transfer	H1b	0.51	0.15	3.40
Emotional identity	Z-direction knowledge transfer	H1c	0.13	0.10	1.30
Behavioral identity	X-direction knowledge transfer	H2a	0.27	0.09	3.00
Behavioral identity	Y-direction knowledge transfer	H2b	0.48	0.12	4.00
Behavioral identity	Z-direction knowledge transfer	H2c	0.34	0.10	3.40
X-direction knowledge transfer	Personal creativity	H3a	0.28	0.09	3.11
Y-direction knowledge transfer	Personal creativity	H3b	0.43	0.13	3.24
Z-direction knowledge transfer	Personal creativity	H3c	0.20	0.12	1.67

In the model test, various fitting indicators was obtained at the same time. If the relevant indicators meet the expected criteria, the model is considered to have a high degree of fit and does not need to be revised. In this study, the values of Chi square/degree of freedom (CMIN/DF) = 1.71, RMSEA = 0.044, CFI = 0.922, NFI = 0.975, IFI = 0.910, and GFI = 0.936 showing that the fitting indicators approach the optimal value, indicating that the model fits well.

## Discussion and Conclusion

The purpose of this study was to examine the mechanism by which the core value identity of knowledge-based professionals influences their personal creativity in a knowledge economy. According to the test results, from the macroscopic point of view, knowledge talents’ core value identity has a certain role in promoting the transfer of tacit knowledge, promoting personal creativity. The value orientation of knowledge talents has an intrinsic incentive to the tacit knowledge transfer behavior. It is an intrinsic feature of the knowledge economy, and it has positive reference significance for the development of human knowledge capital. It is a factor that cannot be ignored in the cultivation of knowledge talents. Therefore, the authors designed and empirically tested a research model. Additionally, unlike other researches that sees knowledge transfer as a consequence of creativity (Bartol and Srivastava, 2002; He et al., 2013), this study is the first to call attention to the way core value identity combined with tacit knowledge transfer (X, Y, and Z-direction) leads in personal creativity.

To postulate the impact of knowledge-based professionals’ core value identity on knowledge transfer, this study categorizes the core value identity as emotional and behavioral identities. The authors used the X, Y, and Z-direction of knowledge transfer in this study, which eventually results in personal creativity of the knowledge-based professionals in China. The overall results of this study relevant to the prior literature (Käser and Miles, 2002; He et al., 2013; Kucharska and Rafał, 2016; Lee, 2018; Abdelwhab Ali et al., 2019; Men et al., 2019), supporting the authentication of these results.

According to the test results, emotional identity has a promoting effect on X-direction knowledge transfer and Y-direction knowledge transfer, but it lacks a promoting effect on Z-direction knowledge transfer. The findings indicate that knowledge-based professionals’ emotional identities exhibit a high degree of identification for core values, ideas, and the ability to perform effective knowledge transfer inside the organization. As a result, H1a, and H1b are accepted, however, H1c is not acceptable. Similarly, this study established that knowledge-based professionals’ behavioral identities affect positively and significantly to X-direction knowledge transfer, Y-direction knowledge transfer, and Z-direction knowledge transfer. It explains how behavioral identity has developed into a critical component of core value identity, affecting knowledge transfer within the organization. As a result, H2a, H2b, and H2c are all acceptable.

X-direction and Y-direction knowledge transfer promotes personal creativity growth, while Z-direction knowledge transfer does not positively affect personal creativity as proved in this study. As creativity required resources such as time, materials, collaboration, and a high level of mental energy to accomplish a task in an organization. Knowledge is a critical source of personal creativity, and information sharing among employees is critical for personal creativity (Men et al., 2019). Based on the findings of this study, we have approved H3a and H3b, but not H3c.

Concluding the overall results of this study it can be stated that cultivation of intellectual talents’ creativity and the incentives for knowledge-based professionals in various institutions in China should follow the following path: ① Pay attention to knowledge-based professionals’ value orientation to guide their recognition of core competencies values. It is a crucial move. ② Because emotional identity has a lack of promotion effect on Z-direction knowledge transfer, it is necessary to guide knowledge-based professionals to pay attention to society and fully demonstrate the social significance of core values. ③ Because Z-direction knowledge transfer lacks promotion effect on personal creativity, it indicates that knowledge-based professionals’ social talent is insufficient, and society’s understanding is not enough. Therefore, the interaction between knowledge-based professionals and society should be strengthened to fully realize the collapse of links. ④ In the actual development of human capital, there should be no evasive attitude toward the values issue, and the core values should be integrated into the corporate culture.

## Research Implications and Future Directions

### Practical Implications

*Seeing is believing; hearing is false.* At present, many knowledge-based professionals do not have a high degree of recognition of core values. Therefore, effectively guiding or enhancing their recognition of core values is an important mission of various institutions in a knowledge economy era. Given the critical nature of tacit knowledge as an organizational resource, managers must understand better how to maintain its preservation. Knowledge-based professionals in today’s Chinese management context have their distinct characteristics; complex material needs, spiritual needs, and values are core elements of spiritual needs. The presentation of socialism’s core values certainly benefits society, the country, and the nation, and it can inspire people and promote uprightness. However, if the core values truly create an intrinsic incentive for knowledge-based professionals, it is necessary to make the core values.

In practice, this research provides managers with a broader view, notably the ability of the two functions to share tacit knowledge and the organization’s ability to manage stakeholders better, improve overall performance, and position itself among closely connected competitors. Improving cognition and comprehending relational dynamics in the firm and team could boost tacit knowledge exchange. As a result, the research has important management implications for creating an organizational intellectual method of tacit knowledge transfer in all directions. The core values identity (emotional and behavioral) has a substantial impact on knowledge transfer, managers should consider deploying better knowledge management platforms to improve the organization’s core values identity. Furthermore, tacit knowledge transfer is a crucial tool for a company to stay ahead of the competition. Managers should concentrate on developing a robust tacit knowledge transmission mechanism.

### 5.2 Theoretical Implications

This research makes several significant advances to the current literature on knowledge transfer and knowledge-based economies. First, this research extends the previous work through theoretical development and empirical testing of the proposed model. The specific development of core value identity (emotional identity and behavioral identity) provides useful tools for future scientific research to explore specific mechanisms of tacit knowledge transfer. Second, China has slipped from one extreme to the other on the value of personal creativity. All of them reflect the irrationality, self-love, and lack of intellectual talent motivation in China. In the nearly 30 years before the reform and opening, the motivation of knowledge-based talents was completely placed on value incentives, values were regarded as a panacea, and material incentives were regarded as Viper beasts. In the 30 years after the reform, material incentives were placed in high status and did not care about value incentives. Neither of these two types of deviant incentives can promote the ultimate creativity of our intellectual talents. Therefore, this research contributes to a more comprehensive understanding of these two types, so supplementing the existing literature.

### 5.3 Limitations and Future Direction

In addition to the implications of this study, some limitations and future directions are also pointed out. In this research, the authors examine the emotional identity and behavioral identity as the core value identity of knowledge professionals. There is still room for expansion of core value identity in promoting the tacit knowledge transfer and personal creativity. By expanding this research framework, future researchers can explore the concept of core value identity in various dimensions. In addition, this study only applies the model to professionals in mainland China, when compared to developed nations, the population of developing countries is generally considered to be more collectivist and less innovative. Therefore, future researchers may apply the model to professionals from a variety of cultural backgrounds to see whether the research findings are unique to that cultural background or replicated across all cultural backgrounds. Finally, we have measured the connection between core value identity, tacit knowledge transfer, and personal creativity. However, future research can contribute to the impact of tacit knowledge transfer on different creativity such as organizational creativity.

## Data Availability Statement

The raw data supporting the conclusions of this article will be made available by the authors, without undue reservation.

## Ethics Statement

The studies involving human participants were reviewed and approved by the Jiangsu University. Written informed consent for participation was not required for this study in accordance with the national legislation and the institutional requirements.

## Author Contributions

YZ: conceptualization, software, writing—original draft preparation, supervision, and funding acquisition. YZ and FS: methodology. YZ and CW: data curation and project administration. CW, MM, and FS: validation. YZ and MM: formal analysis. YZ, CW, FS, and MM: writing—review and editing. All authors have read and agreed to the published version of the manuscript.

## Conflict of Interest

The authors declare that the research was conducted in the absence of any commercial or financial relationships that could be construed as a potential conflict of interest.

## Publisher’s Note

All claims expressed in this article are solely those of the authors and do not necessarily represent those of their affiliated organizations, or those of the publisher, the editors and the reviewers. Any product that may be evaluated in this article, or claim that may be made by its manufacturer, is not guaranteed or endorsed by the publisher.
